# Regional citrate anticoagulation ensures safe and effective kidney replacement therapy in metformin-associated lactic acidosis

**DOI:** 10.1093/ckj/sfaf286

**Published:** 2025-09-11

**Authors:** Francesca Di Mario, Giuseppe Regolisti, Maria Chiara Pacchiarini, Benedetta Mordà, Paolo Greco, Caterina Maccari, Tommaso Di Motta, Vincenzo Oliva, Chiara Italiano, Teresa Coccini, Elisa Roda, Enrico Fiaccadori

**Affiliations:** UO Nefrologia, Azienda Ospedaliero-Universitaria Parma, Dipartimento di Medicina e Chirurgia, Università̀ di Parma, Parma, Italy; Scuola di Specializzazione in Nefrologia, Università di Parma, Dipartimento di Medicina e Chirurgia, Parma, Italy; UO Clinica e Patologia Medica, Azienda Ospedaliero‑Universitaria Parma, Dipartimento di Medicina e Chirurgia, Università di Parma, Parma, Italy; UO Nefrologia, Azienda Ospedaliero-Universitaria Parma, Dipartimento di Medicina e Chirurgia, Università̀ di Parma, Parma, Italy; UO Nefrologia, Azienda Ospedaliero-Universitaria Parma, Dipartimento di Medicina e Chirurgia, Università̀ di Parma, Parma, Italy; UO Nefrologia, Azienda Ospedaliero-Universitaria Parma, Dipartimento di Medicina e Chirurgia, Università̀ di Parma, Parma, Italy; UO Nefrologia, Azienda Ospedaliero-Universitaria Parma, Dipartimento di Medicina e Chirurgia, Università̀ di Parma, Parma, Italy; UO Nefrologia, Azienda Ospedaliero-Universitaria Parma, Dipartimento di Medicina e Chirurgia, Università̀ di Parma, Parma, Italy; UO Nefrologia, Azienda Ospedaliero-Universitaria Parma, Dipartimento di Medicina e Chirurgia, Università̀ di Parma, Parma, Italy; Laboratorio di Immunopatologia Renale “Luigi Migone”, Università degli Studi di Parma, Parma, Italy; Laboratory of Clinical and Experimental Toxicology, Pavia Poison Centre – National Toxicology Information Centre, Toxicology Unit, Istituti Clinici Scientifici Maugeri IRCCS, Pavia, Italy; Laboratory of Clinical and Experimental Toxicology, Pavia Poison Centre – National Toxicology Information Centre, Toxicology Unit, Istituti Clinici Scientifici Maugeri IRCCS, Pavia, Italy; UO Nefrologia, Azienda Ospedaliero-Universitaria Parma, Dipartimento di Medicina e Chirurgia, Università̀ di Parma, Parma, Italy; Scuola di Specializzazione in Nefrologia, Università di Parma, Dipartimento di Medicina e Chirurgia, Parma, Italy

**Keywords:** acute kidney injury (AKI), metformin-associated lactic acidosis (MALA), regional citrate anticoagulation (RCA), sustained low-efficiency dialysis (SLED)

## Abstract

**Background:**

Metformin-associated lactic acidosis (MALA) is a rare but potentially life-threatening complication of metformin therapy, often associated with acute kidney injury (AKI). Sustained low-efficiency dialysis (SLED) offers both haemodynamic stability and effective metformin clearance. However, during regional citrate anticoagulation (RCA), concerns may arise regarding citrate accumulation due to metformin-induced impairment of cellular metabolism. This study assesses the efficacy and safety of SLED with RCA in the management of MALA, providing direct measurements of serum metformin and citrate levels to evaluate drug clearance and potential citrate accumulation.

**Methods:**

A prospective observational study was conducted on consecutive critically ill patients with severe AKI and suspected MALA undergoing a 16-h SLED session with RCA. Demographic, clinical and laboratory data were collected. Serum metformin and citrate levels were measured via high-performance liquid chromatography and enzymatic spectrophotometric analysis, respectively. Mixed effects linear models were used to analyse serum metformin, lactate, citrate, electrolytes and acid–base parameters over time.

**Results:**

Twenty-three patients (median age 79 years; APACHE II score 29) had metformin levels at intensive care unit (ICU) admission above the therapeutic range (median 29.1 mg/l; normal: 0.5–3). ICU mortality was 22% (*n* = 5). SLED led to significant haemodynamic and acid–base improvements, with a marked reduction in serum lactate. Metformin levels decreased from 27.75 mg/l at SLED start to 3.95 mg/l, with minimal rebound. Four SLED sessions (17%) were interrupted, with one being due to impending circuit clotting. No biochemical or clinical signs of citrate accumulation were observed, with serum citrate levels consistently <0.5 mmol/l. No significant correlation was found between serum metformin and citrate levels or between lactate and citrate.

**Conclusions:**

SLED with RCA is safe and effective in patients with MALA, ensuring adequate KRT duration, rapid metformin clearance and acid–base status restoration. Direct citrate measurements confirmed the absence of accumulation, reinforcing RCA as a viable anticoagulation strategy in this clinical setting.

KEY LEARNING POINTS **What was known:**

- Metformin-associated lactic acidosis (MALA) is an infrequent yet potentially fatal adverse effect of metformin therapy, predominantly observed in patients with type 2 diabetes mellitus and concurrent acute kidney injury (AKI). Clinically, it is characterized by multiorgan dysfunction with gastrointestinal symptoms and profound type B lactic acidosis. Early recognition and immediate therapeutic intervention are critical due to the high associated mortality.
- Owing to its pharmacokinetic properties, metformin is amenable to extracorporeal clearance, rendering kidney replacement therapy (KRT) the cornerstone of therapeutic intervention. Sustained low-efficiency dialysis, a hybrid KRT modality, has demonstrated efficacy in enhancing metformin removal and correcting acid–base disturbances, while ensuring haemodynamic stability.
- Regional citrate anticoagulation (RCA) is the preferred anticoagulation strategy in prolonged KRT, thanks to its favourable risk–benefit profile compared with systemic heparin. Nonetheless, its use in MALA remains controversial due to the theoretical risk of citrate accumulation resulting from metformin-induced impairment of mitochondrial oxidative metabolism.

**This study adds:**

- This study provides the first direct evidence supporting the safety and efficacy of RCA in critically ill patients with MALA undergoing KRT. Indeed, by performing parallel and contemporaneous measurements of serum citrate and metformin concentrations, this investigation offers novel insights into the pharmacokinetics and metabolic safety of citrate in the context of impaired mitochondrial function associated with metformin toxicity.
- The findings challenge previous theoretical concerns regarding citrate accumulation in MALA and support the broader applicability of RCA beyond its conventional use in continuous KRT.
- The data generated may contribute to the development of evidence-based guidelines for the most adequate dialysis modality and anticoagulation strategies in the extracorporeal management of MALA.

**Potential impact:**

- The application of our simplified citrate protocol for SLED in patients with MALA may be a useful tool for clinicians to use RCA in this high-risk clinical scenario and according to different local practices.
- The results provide reassurance regarding the metabolic safety of citrate in the context of mitochondrial dysfunction, which may lead to broader implementation of RCA protocols in intensive care unit patients, even in complex metabolic disturbances.
- Finally, this study has the potential to optimize patient outcomes by reducing the risk of haemorrhagic complications associated with systemic anticoagulation while ensuring effective drug clearance and acid–base correction.

## INTRODUCTION

Metformin-associated lactic acidosis (MALA) is a rare but potentially life-threatening complication of metformin therapy, primarily occurring in patients with impaired kidney function [1]. The accumulation of metformin in patients with severe acute kidney injury (AKI) may lead to a peculiar form of multiple organ dysfunction syndrome (MODS), typically presenting with gastrointestinal symptoms and severe type B metabolic lactic acidosis [2, 3]. Metformin is a widely used oral hypoglycaemic agent traditionally recommended as first-line therapy for type 2 diabetes mellitus (T2DM) [4]. Its primary hypoglycaemic effect is due to enhanced peripheral glucose uptake and hepatic gluconeogenesis inhibition [5]. However, the drug interferes with lactate metabolism by impairing its clearance through the inhibition of pyruvate utilization via gluconeogenesis. In fact, it enhances lactic acid production in insulin-dependent tissues by suppressing mitochondrial oxidative phosphorylation and decreasing the mitochondrial redox state [6, 7]. Metformin is administered orally, with a bioavailability of 50–60%. It is a low molecular weight molecule (165 Da), with negligible protein binding and a relatively large volume of distribution (1–5 l/kg), showing significant intracellular distribution [8, 9] (Table 1). Metformin is primarily eliminated by the kidneys, with a clearance that can exceed 500 ml/min, proportionally decreasing with renal function decline [10]. Based on these premises, dose adjustments are recommended for patients with chronic kidney disease (CKD), and metformin body clearance is significantly impaired in patients with AKI (Table 1) [11].

**Table 1:** Physicochemical and pharmacokinetic data of metformin with dose adjustment recommendations in patients with impaired kidney function.

Pharmacokinetic parameters	Values
Molecular weight (Da)	165
Protein binding (%)	<3
Volume of distribution (l/kg)	3 (1–5)
Oral bioavailability (%)	50–60
Time to peak concentration (hours)	1–3 (immediate release)–6–8 (extended release)
Half-life (hours)	1.5–4.9 (plasma)–17.6 (blood)
Endogenous clearance (therapeutic use, normal GFR) (ml/min)	400–650
Therapeutic dose (mg/day)	1500–2550
Therapeutic concentration range (mg/l)	0.5–3
Lethal plasma concentration (mg/l)	>50
Toxic dose for adults (mg/day)	>5
Kidney function	Usual recommended dose
CrCl ≥60 ml/min/1.73 m^2^	No dosage adjustment needed
CrCl 59–45 ml/min/1.73 m^2^	Reduce the dose to 1000 mg daily in cases of increased risk of MALA^a^
CrCl 44–30 ml/min/1.73 m^2^	Reduce the dose to 1000 mg daily
CrCl <30 ml/min/1.73 m^2^	Contraindicated

CrCl: creatinine clearance.

^a^Intended as the presence of comorbidities that imply an increased risk of hypoperfusion and/or hypoxemia.

Modified from Calello et al. [3] and de Boer [11].

The clinical management of MALA requires rapid recognition and intervention, as it is associated with high mortality rates, particularly if left untreated [12]. Given its pharmacokinetic properties (Table 1), the prompt start of kidney replacement therapy (KRT) represents a cornerstone in the complex clinical management of patients with MALA [3, 13]. Currently, definitive evidence regarding the timing, modality and duration of KRT is lacking. While conventional intermittent haemodialysis (IHD) is often preferred for its high efficiency in drug removal and rapid acid–base correction, it cannot prevent significant post-treatment rebound. Conversely, continuous KRT (CKRT), which may be more suitable for haemodynamically unstable patients, often provides inadequate drug clearance with the recommended dialysis dose of 25–30 ml/kg/h. In the past, our group has extensively analysed the efficacy of sustained low-efficiency dialysis (SLED) for treating patients with MALA, supporting the role of this KRT modality in quickly removing the drug while restoring acid–base parameters, maintaining haemodynamic stability and reversing clinical symptoms [14, 15]. As widely observed in general in AKI patients [16], as well as in the specific cohort of patients with MALA, the use of regional citrate anticoagulation (RCA) enabled the prescribed duration to be achieved in 16-h SLED sessions, while minimizing the risk of haemorrhagic complications and reducing treatment downtime [14, 15].

The use of citrate as a circuit anticoagulant has been repeatedly shown to have several advantages over other conventional anticoagulants, such as systemic heparin, in critically ill patients with AKI requiring CKRT or prolonged intermittent KRT (PIKRT) [17]. By restricting anticoagulant activity to the extracorporeal circuit, RCA markedly reduces the risk of systemic haemorrhagic complications and blood transfusions needs [17], which are notably prevalent in AKI, a condition characterized by an increased bleeding risk [18]. Furthermore, RCA has been consistently associated with prolonged filter lifespan, thereby enhancing the efficiency and continuity of KRT with anticoagulant-free protocols, frequently complicated by premature filter clotting, increased blood loss, treatment interruptions, increased nursing workload and increased costs [19]. Despite its widespread endorsement as the preferred anticoagulation modality for PIKRT and CKRT [20, 21], concerns remain regarding the use of citrate in patients with metformin intoxication. Indeed, metformin-induced impairment of aerobic oxidative cellular metabolism may in theory increase the risk of citrate accumulation, potentially leading to electrolyte imbalances and further acid–base disturbances.

On these grounds, this study sought to evaluate the safety and efficacy of the SLED-RCA protocol in the management of critically ill patients with MALA requiring KRT by evaluating acid–base balance, lactate concentrations and serum metformin levels during and following the SLED session, accompanied by concurrent monitoring of serum citrate levels to detect any evidence of citrate accumulation.

## MATERIALS AND METHODS

### Patients

We conducted a prospective observational study between March 2022 and August 2024 involving a cohort of critically ill patients with severe AKI and suspected MALA who underwent 16-h SLED sessions with RCA at the renal intensive care unit (ICU) of Parma University Hospital. The study population included all critically ill patients >18 years of age with severe AKI or AKI in CKD [20], along with a strong clinical suspicion of MALA [3]. Specifically, MALA was defined as a serum lactate concentration greater than 5 mmol/L and an arterial pH less than 7.35 in the context of metformin exposure, in the absence of other predominant causes of lactic acidosis at the time of diagnosis, or when clinical judgment deemed metformin exposure to have significantly contributed to the acid–base disturbance [3]. All participants received supportive care alongside treatments targeting coexisting medical conditions. The study was conducted in compliance with the Declaration of Helsinki and informed consent was obtained from either the patient or a close relative. The study was approved by the local ethics committee (protocol no. 46489).

### Dialysis prescription

Within 1–2 h of admission to the Emergency Department, a 16-h SLED session with RCA was performed with Surdial X Nipro machines and a polysulfone haemofilter (surface 2.1 m^2^, K_Uf_ 27 ml/h/mmHg; Elisio 21M, Nipro, Biwako, Japan) or 5008 CorDiax Fresenius with polysulfone haemofilter (surface 2.2 m^2^, K_Uf_ 68 ml/h/mmHg; FX100, Fresenius, Isola della Scala, Italy), according to availability. Vascular access was obtained via cannulation of the internal jugular, femoral or subclavian vein (right or left side) using a 12-Fr dual-lumen polyurethane catheter (Arrow International, Reading, PA, USA). The dialysis treatment was prescribed according to our institutional protocol [15], by setting the blood flow rate (Qb) at 200 ml/min and dialysis fluid rate (Qd) at 300 ml/min (dialysis bicarbonate and potassium concentration 32 and 4 mmol/l, respectively). Anticoagulation of the extracorporeal circuit was achieved with a high-concentration citrate solution, i.e. the anticoagulant citrate dextrose solution A (ACD-A, 0.8% citric acid, 2.2% trisodium citrate; Fresenius), infused in predilution at 350 ml/h, aiming at an estimated citrate concentration in the haemofilter of 2.5 mmol/l. Since the protocol included a calcium-containing dialysis fluid (Ca^2+^ 1.5 mmol/l), routine calcium infusion was not included in the protocol unless systemic ionized calcium (s-Ca^2+^) was <0.9 mmol/l or circuit ionized calcium (c-Ca^2+^) was <0.6 mmol/l. In these cases, calcium gluconate (10%) (elemental calcium 0.24 mmol/ml) was infused through a separate central venous line at 5 ml/h. Impaired citrate metabolism was suspected in any case of systemic ionized hypocalcaemia (s-Ca^2+^ <0.9 mmol/l) despite calcium supplementation, accompanied by a progressive increase of calcium supplementation requirements and worsening metabolic acidosis. In such cases, an urgent assessment of the calcium ratio (total calcium:s-Ca^2+^ ratio) was scheduled and a value >2.5 was considered an indirect indicator of citrate accumulation, definitely confirmed when associated with a significant increase in serum citrate levels [22]. Treatment monitoring included measuring activated clotting time (ACT; Hemochron Jr Signature Elite, ITC, Edison, NJ, USA) and blood gas analysis (BL800 Flex blood gas analyser; Radiometer Medical, Copenhagen, Denmark).

### Laboratory monitoring

Complete laboratory workup was carried out at baseline and on the day subsequent to the SLED session. Phosphate and magnesium losses with KRT were replaced, when needed, by intravenous administration of sodium glycerophosphate pentahydrate (Glycophos 20 mmol/20 ml, Fresenius Kabi Norge AS, Halden, Norway) and magnesium sulphate, respectively.

As detailed in the Supplemental Material, blood samples for metformin and citrate serum levels were collected from the arterial line during and after the SLED session.

### Statistical analysis

Data were presented as mean ± standard deviation (SD) for normally distributed continuous variables, median and interquartile range (IQR) for non-normally distributed variables and frequencies for categorical variables at the start of the SLED session. Serum metformin, lactate and citrate trajectories during and up to 8 h after the session were analysed using mixed effects linear models, with time points as fixed factors and clinical scores [Acute Physiologic and Chronic Health Evaluation II (APACHE II), Model for End-Stage Liver Disease (MELD), Sequential Organ Failure Assessment (SOFA), Charlson Comorbidity Index (CCI)] and baseline estimated glomerular filtration rate (eGFR) as covariates. Patients were treated as a random effect. Additionally, sodium, potassium, bicarbonate, s-Ca^2+^, activated clotting time and transmembrane pressure (TMP) during SLED were analysed similarly. Linear regression examined the relationships between metformin and citrate, and lactate and citrate, adjusting for relevant covariates. Statistical significance was defined as *P* < .05 and analyses were conducted using SPSS Statistics 28.0 (IBM, Armonk, NY, USA).

## RESULTS

### Patients’ condition at renal ICU admission

During the study period, 24 patients were diagnosed with a clinical presentation consistent with MALA accompanied by severe AKI and were therefore treated urgently with a 16-h SLED session, according to our department protocol. Upon data analysis, serum metformin levels at ICU admission in one patient were inconsistent with metformin intoxication (0.6 mg/l), thus this patient was excluded from the analysis, resulting in a final study population of 23 patients.

The demographic, clinical and biochemical characteristics of the patients at ICU admission are described in Table 2. The median age was 79 years (IQR 74–83), with a predominance of females [*n* = 16 (70%)]. The median APACHE II score was 29 (IQR 24–34). At admission, all patients were markedly hypotensive, with a mean SOFA score of 8.4 (SD 1.5). More than half of the patients required vasopressor support with norepinephrine (infusion rate range 0.05–1.47 μg/kg/min) and four patients (17%) required non-invasive mechanical ventilation. Oligoanuria was present in all patients and all patients presented with stage 3 AKI, according to the most recent Kidney Disease: Improving Global Outcomes criteria [20]. At ICU admission, sepsis was identified in the majority of patients [*n* = 19 (83%)] and was regarded as the principal predisposing factor for the development of AKI, in conjunction with hypovolaemia and ischaemic acute tubular necrosis due to severe kidney hypoperfusion. Additionally, the concomitant use of nephrotoxic agents, specifically non-steroidal anti-inflammatory drugs, was documented in two patients. The median baseline eGFR [by the Chronic Kidney Disease Epidemiology Collaboration (CKD-EPI) equation] was 65.5 ml/min/1.73 m^2^ (IQR 53.1–82.9), with a mean baseline outpatient serum creatinine of 0.9 mg/dl (SD 0.3). Ten patients (43%) had pre-existing CKD, classified as stage G3a in six cases and stage G3b in four, with a median baseline eGFR_CKD-EPI_ of 48.7 ml/min/1.73 m^2^ (IQR 41.1–55.6). One patient had a history of hepatitis C virus–related chronic liver disease with Child–Pugh B liver cirrhosis, while nine patients (39%) had an acute episode of liver dysfunction as a component of MODS, with a MELD score ≥25. The median overall MELD score was 23 (IQR 21–26) (Table 2).

**Table 2:** Demographic and clinical characteristics at ICU admission.

Variable	Values
Age (years), median (IQR)	79 (74–84)
Male, *n* (%)	7 (30)
APACHE II score, median (IQR)	29 (24–34)
SOFA score, mean (SD)	8.4 (1.5)
MELD score, median (IQR)	23 (21–26)
Mechanical ventilation, *n* (%)	4 (17)
Vasopressors use for haemodynamic instability, *n* (%)	16 (70)
Sepsis, *n* (%)	19 (83)
Chronic comorbidities, *n* (%)	
Arterial hypertension	19 (83)
Coronary arteries disease	3 (13)
Heart failure	2 (8.6)
CKD	10 (43)
COPD	5 (22)
Chronic liver disease	1 (4)
Charlson Comorbidity Index, mean (SD)	6 (1.6)
BMI (kg/m^2^), median (IQR)	24.1 (21–28.6)
Baseline renal function (eGFR_CKD-EPI_) (ml/min), median (IQR)	65.5 (53.1–82.9)
Usual metformin dose (mg/day), mean (SD)	1950 (550)
Serum creatinine (mg/dl), mean (SD)	7.2 (3)
BUN (mg/dl), median (IQR)	92 (61–109)
Haemoglobin (g/dl), median (IQR)	11.5 (10.2–12.6)
Haematocrit (%), mean (SD)	37 (7)
Platelets (×10^3^/μl), median (IQR)	328 (247–407)
White blood cells (×10^3^/μl), median (IQR)	17.27 (11.29–22.84)
Glucose (mg/dl), median (IQR)	148 (96–232)
Sodium (mmol/l), mean (SD)	135 (6.6)
Potassium (mmol/l), mean (SD)	5.9 (1.37)
Calcium (mg/dl), mean (SD)	8.5 (0.9)
AST (UI/l), median (IQR)	24 (19–58)
ALT (UI/l), median (IQR)	23 (11–28)
Total bilirubin (mg/dl), mean (SD)	0.6 (0.3)
INR, median (IQR)	1.1 (1–1.5)
aPTT ratio, median (IQR)	0.95 (0.8–1)
Albumin (g/dl), mean (SD)	2.9 (0.5)
pH, mean (SD)	6.99 (0.15)
PaO_2_ (mmHg), median (IQR)	106 (95–125)
PaCO_2_ (mmHg), median (IQR)	19 (15–26)
Lactate (mmol/l), mean (SD)	15.6 (1.50)
Bicarbonate (mmol/l), mean (SD)	5.8 (3.8)
Anion gap (mmol/l), mean (SD)	35 (9)
Length of ICU stay (days), median (IQR)	3 (1.5–6)
Death in the ICU, *n* (%)	5 (22)

COPD: chronic obstructive pulmonary disease; BMI: body mass index; BUN: blood urea nitrogen; AST: aspartate aminotransferase; ALT: alanine aminotransferase; INR: international normalized ratio; aPTT: activated partial thromboplastin time.

At ICU admission, the arterial blood gas analysis showed severe high anion gap metabolic acidosis with acidaemia and hyperlactataemia in all patients [mean pH 6.99 (SD 0.15), HCO_3_^−^ 5.8 mmol/l (SD 3.8), anion gap 35 (SD 9), lactate 15.6 mmol/l (SD 1.5)].

In all included patients the diagnosis of MALA was confirmed subsequently, with median serum metformin concentrations at ICU admission of 29.1 mg/l (IQR 13–39).

### Clinical course and acid–base status during and after SLED

As detailed in the Methods section, KRT was initiated specifically for MALA as the primary clinical indication. The primary site of central venous catheter (CVC) insertion was the right internal jugular vein [*n* = 14 (61%)], followed by the left internal jugular vein in 8 patients (35%) and the right femoral vein in 1 patient (4%), with catheter lengths ranging from 16 to 25 cm based on the insertion site and individual anatomical characteristics. The mean prescribed weight gain during the 16-h treatment was 1450 ml (SD 900), while the actual increase was 1960 ml (SD 1610). Five patients (21%) required blood transfusions at the start of dialysis treatment, with a mean total volume of 400 ml (SD 136). In 21 of 23 patients we observed a gradual improvement in haemodynamic status during dialysis treatment, allowing for a progressive reduction in norepinephrine dose, with a parallel increase in urine output. In contrast, two patients experienced a rapid decline in hemodynamic and respiratory conditions during treatment, leading to premature discontinuation of the SLED session (after 13 and 14 h). Both patients died within 24 h of initiating dialysis, with mortality being attributed primarily to the progression of underlying critical illness and multi-organ failure. Specifically, acute intestinal ischaemia was the primary suspected cause of death in one patient, while refractory septic shock, confirmed by positive blood cultures for *Escherichia coli*, was identified in the other. In all 23 patients, acid–base balance parameters and lactate levels improved significantly during the dialysis session (*P* < .001), without any further increase in the hours following its completion (Table 3). All surviving patients were dialysis-free at the time of hospital discharge. The median ICU stay was 3 days (IQR 1.5–6), while the median total hospital stay was 12 days (IQR 7–26). The ICU mortality rate was 22% (*n* = 5): as mentioned above, two patients died during dialysis treatment and three within 72 h after the end of KRT (in two cases intestinal ischaemia, in one severe respiratory failure).

**Table 3:** Acid–base status, electrolytes and intradialytic variables related to RCA at SLED start and during the dialysis session and serum metformin, lactate and citrate concentration during and up to 8 h after SLED end.

Variable	SLED start	SLED 2 h	SLED 8 h	SLED 12 h	SLED 16 h	1 h after	4 h after	8 h after	*P*-value
ACT (seconds)	123.6 (9.30)	121.7 (7.45)	113.9 (5.16)	115.2 (3.98)	117.5 (4.90)	NA	NA	NA	.504
s-Ca^2+^ (mmol/l)	1.11 (0.02)	1.11 (0.02)	1.12 (0.01)	1.13 (0.02)	1.11 (0.02)	NA	NA	NA	.869
c-Ca^2+^ (mmol/l)	NA	0.98 (0.22)	NA	NA	NA	NA	NA	NA	NA
HCO_3_^−^ (mmol/l)	9.9 (0.98)	13.1 (1.04)	18.1 (0.75)	20.3 (0.47)	20.5 (0.38)	NA	NA	NA	<.001
K^+^ (mmol/l)	5.2 (0.2)	4.6 (0.2)	4.5 (0.1)	4.4 (0.1)	4.3 (0.1)	NA	NA	NA	<.001
Na^+^ (mmol/l)	138.8 (1.1)	139.2 (1.0)	139.3 (1.0)	137.3 (0.7)	137.9 (0.6)	NA	NA	NA	.414
Lactate (mmol/l)	15.6 (1.50)	10.9 (1.01)	5.2 (0.87)	3.0 (0.42)	2.5 (0.34)	2.6 (0.47)	2.4 (0.37)	1.9 (0.33)	<.001
Calcium ratio	1.91 (0.22)	NA	NA	NA	NA	NA	NA	NA	NA
TMP (mmHg)	42.9 (7.48)	44.3 (7.59)	48.4 (8.26)	45.4 (7.87)	37.5 (6.92)	NA	NA	NA	.608
Metformin (mg/l)	27.75 (3.21)	16.74 (2.01)	8.90 (1.29)	5.59 (0.58)	3.95 (0.50)	5.01 (0.64)	5.12 (0.61)	5.26 (0.48)^a^	<.001
Citrate (mmol/l)	0.14 (0.03)	0.25 (0.04)	0.29 (0.05)	0.31 (0.05)	0.35 (0.05)	0.21 (0.04)	0.15 (0.02)	0.11 (0.01)	<.001

ACT: activated clotting time; HCO_3_^−^: bicarbonate; K^+^: potassium; Na^+^: sodium; NA: not available.

^a^ *P* < .01 in the comparison among rebound values at 1, 4 and 8 h.

Data are presented as estimated marginal mean (standard error). Significance values refer to the average effect of the predictor variable (time during and after the end of the SLED session, expressed in hours). Calcium ratio refers to a total to ionized calcium concentration (s-Ca:s-Ca^2+^).

**Figure 1:**
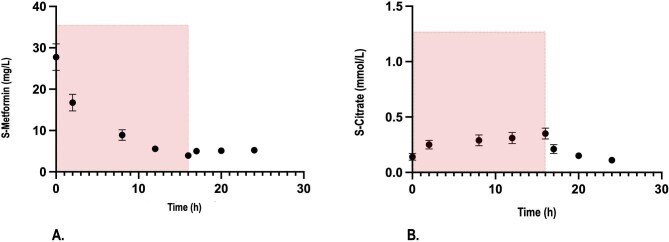
Trend of **(A)** metformin and **(B)** citrate serum concentration during a 16-h SLED session and up to 8 h after the end of the session. Points and vertical bars indicate estimated marginal means and standard errors, respectively, obtained by linear mixed effects models for repeated measures adjusted for relevant covariates (see Methods). The pink box indicates the SLED period.

Serum metformin levels significantly decreased during the SLED session, with a statistically significant but clinically negligible rebound in drug concentration observed after treatment (Table 3, Fig. 1A).

### Safety and efficacy of RCA during SLED

**Figure 2:**
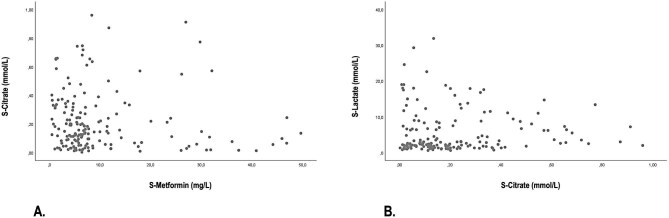
**(A)** Relationship between serum metformin concentration and serum citrate concentration throughout the 16-h SLED session and up to 8 h after the end of the session. **(B)** Relationship between serum lactate concentration and serum citrate concentration throughout the 16-h SLED session and up to 8 h after the end of the session. Data points represent measurements taken at specific time intervals during the session, illustrating the temporal fluctuations of both parameters.

Of the 23 SLED sessions analysed, 4 (17%) were prematurely interrupted, in 2 cases due to patients’ clinical status deterioration, in 1 case due to CVC malfunctioning and in 1 case due to impending circuit clotting. In all cases, full blood volume restitution was performed. As shown in Table 3, TMP did not significantly increase during the SLED sessions and the prescribed treatment duration was attained in more than 80% of the cases. Calcium gluconate infusion was scheduled during three KRT sessions (13%), in one case from SLED start due to s-Ca^2+^ levels <0.9 mmol/l and in two cases from the second hour due to c-Ca^2+^ levels <0.6 mmol/l. As mirrored by the stability of s-Ca^2+^ concentration, the unchanged need for calcium gluconate supplementation and the gradual improvement in acid–base balance, no patient exhibited changes in laboratory parameters consistent with citrate accumulation. Furthermore, serum citrate concentrations showed no clinically significant alterations, although a mild, albeit statistically significant, increase was observed during the SLED session. The mean serum citrate levels remained consistently <0.5 mmol/l, with a progressive reduction after the end of dialysis, with values similar to those measured in other cohorts of critically ill patients with AKI without metformin intoxication treated with RCA-SLED [23–25] (Table 3; Fig. 1B). As confirmation of citrate safety, at regression analysis we found no significant correlation between metformin and citrate serum levels (*P* = .308) or between lactate and citrate serum concentrations during and after the end of SLED (*P* = .401) (Fig. 2A and B). In both patients who died during the SLED session, there was no evidence of citrate accumulation, as indicated by stable serum citrate and ionized calcium levels, unchanged calcium gluconate supplementation and a progressive decrease of serum lactate levels.

Regarding electrolyte monitoring, 16 of 23 sessions (70%) were complicated by hypophosphataemia and 10 (43%) by hypomagnesaemia.

## DISCUSSION

To the best of our knowledge, this is the first study able to demonstrate the safety of citrate as a circuit anticoagulant in critically ill patients undergoing KRT for AKI-associated MALA, by providing a detailed analysis of serum citrate measurements compared with serum metformin concentration during and after a dialysis session.

In recent years, the adoption of a streamlined RCA protocol, optimized for a 16-h SLED session, has progressively become the standard of care in our renal ICU for critically ill patients with MALA. Indeed, thanks to its pharmacokinetic characteristics (Table 1), metformin can be efficiently removed by extracorporeal treatments, and the use of this hybrid dialysis technique provides an optimal balance between the high solute clearance efficiency characteristic of conventional IHD and the haemodynamic stability associated with CKRT. As extensively validated in various clinical settings, citrate anticoagulation facilitates the delivery of the prescribed dialysis dose, while mitigating the risk of bleeding complications and minimizing treatment downtime.

MALA is a rare but potentially fatal complication of metformin treatment, occurring in ≈10 cases per 100 000 individuals per year, with an associated mortality risk ranging from 17% to 50% [26]. The impaired lactate homeostasis characteristic of this condition arises from the drug’s inhibition of mitochondrial respiratory chain complex I, which results in reduced oxidative phosphorylation and enhanced anaerobic glycolysis. This results in reduced hepatic gluconeogenesis, reduced pyruvate metabolism and increased lactate production with impaired lactate clearance. Additionally, metformin impairs the mitochondrial redox balance by increasing the NADH:NAD⁺ ratio, further promoting lactate accumulation [27]. In conditions with renal function impairment, where metformin clearance is reduced, its accumulation exacerbates these effects, leading to severe lactic acidosis [26]. Other contributing factors may include hypoxia, liver dysfunction and conditions that increase lactate production, such as sepsis or heart failure, often coexisting in critically ill patients [28]. Historically, guidelines have recommended against the use of citrate in patients with elevated lactate concentrations due to the increased risk of citrate accumulation in cases of impaired cellular respiration [20]. This implies that patients with the most severe clinical conditions at KRT initiation are exposed to the highest risk of complications associated with systemic heparin anticoagulation, such as an increased bleeding risk and the need for blood transfusions. Hence they may receive no anticoagulation during KRT, with the consequent risk of unexpected KRT interruptions and circuit clotting with associated blood loss.

Citrate is a small molecule (198 Da) available as trisodium citrate salt with or without citric acid in different solutions. The anticoagulant solution is normally infused before the filter to provide chelation of the ionized calcium, thus blocking the coagulation cascade at different steps [29]. Since the calcium–citrate complexes are characterized by elevate diffuse and convective clearances, with a sieving coefficient between 0.85 and 1, 40–60% of the infused citrate is lost in the effluent fluid [30]. The remaining amount returning to the patient’s circulation represents the citrate metabolic load, which is quickly metabolized in the liver and skeletal muscle via the oxygen-dependent Krebs cycle, contributing to bicarbonate generation and energy production [31]. The systemic citrate levels, still considered the gold standard to confirm the diagnosis of citrate accumulation, are representative of whole-body citrate balance, the difference between citrate load and endogenous metabolic clearance [21]. It has been hypothesized that the altered oxidative metabolism characteristic of tissue hypoperfusion, typically revealed by hyperlactataemia, may impair citrate metabolism, resulting in metabolic acidosis, systemic hypocalcaemia and a progressive increase in serum citrate concentration [32]. This concern arises from the assumption that elevated serum lactate, a hallmark of intracellular hypoxia and an anaerobic metabolic state, may predict citrate accumulation [33]. However, recent evidence challenges the predictive value of baseline serum lactate levels for citrate accumulation, suggesting that lactate kinetics may play a more significant role in identifying conditions of ongoing intracellular hypoxia and impaired function of the respiratory chain [34–36].

In a large retrospective study conducted in critically ill patients undergoing continuous venovenous hemodialysis (CVVHD) with RCA, the lactate clearance was found to be a more reliable predictor of citrate accumulation compared with the initially elevated lactate concentration, even among patients with the highest baseline lactate levels [36]. In patients with AKI-associated metformin intoxication, which is the primary trigger of lactic acidosis, the start of a highly efficient KRT enables rapid drug removal. This promotes a shift toward aerobic cellular metabolism and allows adequate citrate metabolism. As shown in Table 3 and Fig. 1, in our series serum metformin and lactate concentrations progressively decreased throughout the SLED session, while citrate serum levels, after a mild increase consistent with the predilution infusion, remained steadily <0.5 mmol/l and decreased after the end of dialysis. In this regard, no significant correlation was found between serum metformin and citrate levels, nor between lactate and citrate concentrations during and after the 16-h SLED session (Fig. 2).

In this context, it is essential to consider that patients with MALA exhibit a highly complex clinical profile characterized by haemodynamic instability, severe metabolic acidosis and frequently concomitant acute liver dysfunction (Table 2). In order to maximize the safety of RCA in patients with potentially severe impairment of citrate metabolism [median MELD score at KRT start 23 (IQR 21–26)], as already reported in other high-risk clinical contexts [37–39], we optimized our standard RCA protocol for SLED [23, 25] with the aim of reducing the metabolic citrate load, by exploiting the high diffusive clearance of this hybrid technique, which significantly mitigates the risk of citrate accumulation. Indeed, the absence of citrate accumulation in our cohort may be partly explained by the high citrate clearance associated with the higher dialysate flow rates used in SLED, which, in the peculiar clinical setting of patients with MALA, facilitated the effective clearance of both metformin and citrate. Furthermore, the use of calcium-containing dialysis fluid, by exploiting the calcium backfiltration, significantly reduces the risk of systemic ionized hypocalcaemia. In contrast, the use of RCA-CKRT in this specific patient population, at the typically recommended doses of 25–30 ml/kg/h, would result in lower instantaneous solute clearance, potentially insufficient for effective metformin and citrate removal.

Using this approach, we did not observe any sign of citrate accumulation during dialysis treatment, while recording a progressive increase of bicarbonate serum levels and no significant changes in s-Ca^2+^ (Table 3). Notably, severe hypocalcaemia never occurred, and the infusion of calcium gluconate was necessary during three KRT sessions only (13%). Hence the use the RCA, even in this peculiar clinical scenario, proved to be a safe and efficacious treatment, which allowed us to reach the prescribed elapsed time in more than 80% of patients. Given the prolonged dialysis duration, the high diffusive solute clearance and the use of a standard haemodialysis machine, close laboratory monitoring is essential to prevent and early diagnose any treatment-related electrolyte disorders, such as hypophosphataemia and hypomagnesaemia, which may complicate the treatment [17, 40, 41]. In this regard, we also utilized a standard dialysis solution containing 4 mmol/l of potassium, aiming for a gradual and controlled correction of hyperkalaemia. This approach helped avoid an abrupt decrease in serum potassium levels that could increase the risk of potentially life-threatening arrhythmias, particularly in critically ill patients with AKI and severe acidosis [42].

This study has limitations. First, it did not include a control group, which limits the ability to compare citrate treatment with alternative therapeutic approaches (e.g. anticoagulation with unfractionated or low molecular weight heparin). However, given the inherent high haemorrhagic risk of critically ill patients with severe AKI [18, 29, 31], ethical concerns would arise in designing studies incorporating a control arm. Second, the limited sample size may hinder generalization of our findings to a broader population. However, a systematic review included only two observational studies of metformin intoxication treated with extracorporeal techniques with maximum sample sizes of 30 and 66 patients [3]. Despite these limitations, we believe that this study provides important insights into the optimization of extracorporeal treatment in patients with MALA. Notably, considering the extreme frailty of this patient population, strategies aimed at reducing metabolic citrate load by decreasing the citrate dose and/or increasing the citrate dialysis clearance, combined with meticulous laboratory monitoring, are mandatory to minimize the risk of citrate accumulation. In this regard the use of SLED may help maintain the haemodynamic stability associated with CKRT while enhancing both metformin and citrate clearance due to its higher solute clearance efficiency (mean effluent volume of 300 ml/min). The close laboratory monitoring should include a regular assessment of s-Ca^2+^ and acid–base parameters with lactate concentrations, careful evaluation of calcium supplementation requirements and attention to any signs of worsening clinical status which may favour citrate accumulation. In case of suspicion, a fast evaluation of the calcium ratio and, if feasible, citrate serum levels will definitely confirm the diagnosis, which requires prompt interruption of this anticoagulation strategy.

By adhering to these practical recommendations, RCA may be safely implemented in patients with AKI-associated MALA, thereby enabling prolonged and effective dialysis treatments and potentially signifying a paradigm shift in the therapeutic management of this complex and rapidly evolving clinical condition.

## CONCLUSIONS

The use of SLED with citrate appears to be a safe and effective therapeutic strategy for patients with MALA and severe AKI. Establishing the safety profile of citrate may facilitate the implementation of prolonged and efficacious dialysis therapy, potentially signifying a paradigm shift in the therapeutic management of this complex and rapidly evolving clinical condition.

## Supplementary Material

sfaf286_Supplemental_File

## Data Availability

The datasets used and/or analysed during the current study are available from the corresponding author upon reasonable request.
