# Supplementary material for: Regional citrate anticoagulation ensures safe and effective kidney replacement therapy in metformin-associated lactic acidosis
Source: Clin Kidney J. 2025 Sep 11;18(10):sfaf286. doi: 10.1093/ckj/sfaf286 (PMC12508805; doi:10.1093/ckj/sfaf286)
Supplement: sfaf286_Supplemental_File [file sfaf286_supplemental_file.docx]

***Serum metformin determination***

In accordance with institutional protocols for suspected Metformin-associated Lactic Acidosis (MALA), for each enrolled patient, blood samples were collected from the arterial line to confirm metformin intoxication, at SLED start, during the SLED session (at 2^nd^, 8^th^, 12^th^, and 16^th^ hours), and then after 1, 4 and 8 hours from the end of dialysis session. The blood samples for metformin measurements were stored at -80°C and subsequently sent to an external specialized facility.

Serum metformin was determined by HPLC (High performance liquid chromatography). The procedure was developed (i.e. sample preparation, sensitivity, accuracy, precision, linearity) in the laboratory of Clinical and Experimental Toxicology, ICRS Maugeri IRCCS Pavia, Pavia, Italy, and then engineered and validated by Eureka Lab Division – Sentinel Company as a commercial kit ready to use, simple, fast and reliable, including all needed materials and reagents to treat the serum and to perform the chromatographic analysis (Eureka Scientific Collection, 2016). The metformin quantification was achieved on a Shimadzu Prominence UFLC system (Shimadzu Corporation, supplied by B.S.N. Srl Biological Sales Network, Castelleone (CR), Italy) which consisted of DGU-20A 5R Degassing Unit, a LC-20AT Isocratic Pump, a SIL-20A HT Autosampler, a CTO-20A Thermostatted Column Compartment, a SPD-20AV UV-VIS Spectrophotometer Detector, and a CBM-20A System Controller. The instrument software (Clarity™) version 3.0.6.589 was employed for the data acquisition, processing, and instrument control in regulated environment.

Briefly, the chromatographic separation of metformin was achieved using a non-porous Agilent Bio SCX column (250×4.6 mm, 10 μm particle size) coupled with a guard column Agilent Bio SCX, NP 10, SS guard (4 x 10 mm, non-porous,10 μm) (B.S.N. Srl Biological Sales Network, Castelleone (CR), Italy). The detection wavelength was 232 nm using a mobile phase consisting of a mixture of 0.05 M buffer solution pH 5.6 at a flow rate of 1.2 ml/min. The separation was achieved with an injection volume of 50 µl at 35 °C with a total run time of 8.0 min. Metformin was eluted with a retention time of 3.2-3.3 min. The LLOQ for metformin was 0.2 μg/ml, and the LLOD was 0.2 μg/ml.

***Serum citrate determination***

For each enrolled patient, blood samples for citrate measurement were also collected from the arterial line, during and after the end of SLED session, in order to assess the eventual citrate accumulation (at SLED start, at 2^nd^, 8^th^, 12^th^, and 16^th^ hours, and then after 1, 4 and 8 hours from the end of dialysis session). These samples were also stored at -80°C and then analysed by using a commercially available ultraviolet test kits for enzymatic spectrophotometric analysis (Enzyplus EZA785+; Biocontrol Systems, Rome, Italy).
